# Seasonal Patterns of Common Respiratory Viral Infections in Immunocompetent and Immunosuppressed Patients

**DOI:** 10.3390/pathogens13080704

**Published:** 2024-08-20

**Authors:** Fotis Theodoropoulos, Anika Hüsing, Ulf Dittmer, Karl-Heinz Jöckel, Christian Taube, Olympia E. Anastasiou

**Affiliations:** 1 Department of Pulmonary Medicine, University Hospital of Essen-Ruhrlandklinik, 45239 Essen, Germany; fotis.theodoropoulos@rlk.uk-essen.de (F.T.); christian.taube@rlk.uk-essen.de (C.T.); 2 Institute of Medical Informatics, Biometry and Epidemiology, University Hospital Essen, University Duisburg-Essen, 45122 Essen, Germany; anika.huesing@uk-essen.de (A.H.); k-h.joeckel@uk-essen.de (K.-H.J.); 3 Institute for Virology, University Hospital Essen, University of Duisburg-Essen, 45147 Essen, Germany; ulf.dittmer@uk-essen.de; 4 Centre for Clinical Studies (ZKSE), Institute for Medical Informatics, Biometry and Epidemiology, Medical Faculty, University Duisburg-Essen, 45122 Essen, Germany

**Keywords:** seasonality, RSV, PIV, influenza, HMPV, immunocompromised

## Abstract

Introduction: Several respiratory viruses have been shown to have seasonal patterns. The aim of our study was to evaluate and compare these patterns in immunocompetent and immunosuppressed patients for five different respiratory viruses. Methods: We performed a retrospective analysis of results for 13,591 respiratory tract samples for human metapneumovirus (HMPV), influenza virus, parainfluenza virus (PIV) and respiratory syncytial virus (RSV) in immunocompetent and immunosuppressed patients. A seasonal pattern was aligned to the data of immunocompetent patients through a logistic regression model of positive and negative test results. Results: A narrow seasonal pattern (January to March) was documented for HMPV. Most RSV infections were detected in the winter and early spring months, from December to March, but occasional cases of RSV could be found throughout the year. The peak season for PIV-3 was during the summer months, and that for PIV-4 was mostly in autumn. A narrow seasonal pattern emerged for influenza virus as most infections were detected in the winter, in January and February. The seasonal patterns of HMPV, RSV, PIV, and influenza virus were similar for both immunocompetent and immunocompromised patients. Conclusions: We found no difference in the seasonality of HMPV, RSV, PIV, and influenza virus infections between immunosuppressed and immunocompetent hosts.

## 1. Introduction

Respiratory tract infections (RTIs) are a major cause of morbidity and mortality. Globally, lower respiratory tract infections (LRTIs) are the fourth most frequent cause of death. This effect is more pronounced in low-income countries, where LRTIs are the second most frequent cause of death, and less pronounced but still very significant in high-income countries, where LRTIs are the sixth most frequent cause of death [1]. In addition to their impact on human health and life, LRTIs have a detrimental effect on the economy, with direct costs associated with health services, disease management, industries, business and trade, education, and indirect costs due to productivity losses [2]. Understanding the seasonal pattern of common respiratory viruses may help us anticipate subsequent high and low seasons and facilitate an optimal allocation of healthcare resources.

Common causes of viral respiratory infection include the influenza virus, with its subtypes A and B, respiratory syncytial virus (RSV), human metapneumovirus (HMPV), parainfluenza virus (PIV), with its subtypes PIV 1, 2, 3, and 4. HMPV has the potential to induce respiratory illnesses in individuals across all age groups. However, it particularly impacts young children, older adults, and individuals with compromised immune systems. The clinical manifestation of HMPV infection includes the usual symptoms of a respiratory infection, ranging from an asymptomatic or mildly upper respiratory infection to severe pneumonia [3,4]. There are four subtypes of PIV. They mostly cause symptoms similar to those of the common cold, usually in small children; however, they can cause a more severe disease such as croup or pneumonia. Different subtypes have been reported to circulate at different times of the year [5]. RSV is a common respiratory virus, usually causing mild “common cold” symptoms, but it can cause serious disease in infants, in the form of bronchiolitis, older adults, or people with chronic conditions [6]. Influenza viruses are the cause of the seasonal flu. While there are four types of influenza viruses (A–D), only A and B cause seasonal epidemics of the disease. Groups with a high risk for adverse outcomes after influenza virus infection include young children, pregnant women, people with chronic medical conditions, and immunosuppressed individuals [7].

RTIs have an even more devastating impact on the health and life of immunosuppressed patients. RTIs in immunocompromised patients have been associated with increased morbidity and mortality compared to immunocompetent hosts [8]. In a study with 1301 immunocompromised patients, the risk of death was 13% for RSV infections, 8% for HMPV infections, and 7% for influenza and PIV infection, while the respective rates were 1.5 to 2 times greater in hematological cases [9]. Immunocompromised patients are at risk for more severe or complicated influenza-induced disease [10]. In a US study with 35,348 adults, immunocompromised patients had higher mortality (adjusted odds ratio [aOR], 1.46; 95% confidence interval [CI], 1.20–1.76), were hospitalized longer (adjusted hazard ratio of discharge, 0.86; 95% CI, 0.83–0.88), and were more likely to require mechanical ventilation (aOR, 1.19; 95% CI, 1.05–1.36) [11]. Previous studies have indicated that patients with hematological malignancies, those who have undergone hematopoietic stem cell transplant (HSCT), or solid organ transplant recipients have a significantly increased risk for severe respiratory syncytial virus (RSV) infection and fatal outcome [12]. Parainfluenza virus (PIV) lower RTI has been found to be a major risk factor for PIV-associated mortality in patients with hematological malignancies and HSCT recipients, irrespective of age, with mortality rates reaching 31% [13].

Many viral RTIs follow a seasonal pattern, which varies in different geographic regions. In temperate climate regions, viral RTIs reach their peak in winter, and in tropical areas, during the rainy season [14,15,16,17]. The fundamental processes behind the seasonal occurrence of respiratory viral infections have been discussed and debated for numerous years. Two primary elements that have been thought to significantly contribute to seasonality are alterations in environmental conditions (for example, temperature and humidity) and human conduct [18]. A previous study from Scotland described that RSV and influenza A have the largest seasonal peaks, appearing in November–December and December–January, respectively, while the high season for influenza B is from February to March, for HMPV in March, and for PIV-3 from April to May. PIV-2 has two peaks, a major peak around October–November and a minor peak in July, while a clear seasonality is not noticeable for HPIV-1 [19]. A German study showed that RSV, influenza A virus, and HMPV peak in the winter months [15]. A Chinese study, evaluating data from different climate zones, demonstrated that RSV and influenza virus have annual peaks in Northern China, but biannual peaks in Southern China, while PIV had higher positive rates in the spring–summer months and HMPV in winter–spring, especially in the north [20].

We have previously described the seasonality of non-SARS, non-MERS coronavirus infections, which followed a seasonal pattern peaking from December to March and plunged from July to September in Germany. Interestingly, we found that the seasonal effect was less pronounced in immunosuppressed patients compared to immunocompetent patients, with “off-season” detection of coronavirus more frequent in immunosuppressed patients [21]. A 1997 study from a USA population of immunocompromised and immunocompetent patients indicated that the patterns of occurrence of viral RTIs by time of year in immunocompromised patients are similar to those described for these viruses among immunocompetent persons, except for the frequency of RSV infection [22].

The aim of our study was to evaluate the seasonality of HMPV, PIV, influenza virus, and RSV infection in immunocompetent and immunosuppressed patients.

## 2. Patients and Methods

Data from the testing of respiratory tract samples (n = 13,591), including nose/throat swabs, saline gurgle samples, tracheal aspirates, and bronchoalveolar lavages, were retrospectively analyzed. The testing took place at the Institute for Virology of the University Hospital Essen, Germany, from June 2013 to December 2019. The samples were tested with the respiratory viral panel (FTD, Siemens, Erlangen, Germany) according to the manufacturer’s instructions, for the detection of human metapneumovirus (HMPV), influenza virus (A and B), parainfluenza virus (PIV 1–4), and respiratory syncytial virus (RSV). The analysis included all tested samples, meaning that a patient could contribute more than once over time, with the following exception. Data were purged from repeated (within 28 days) positive findings of single viruses, because these were interpreted as indicating the same infection case. Repeated negative findings were included in further analyses. Nucleic acid extraction was performed using MagNA pure (Roche, Mannheim, Germany). Patients infected with multiple viruses at the same time contributed to all corresponding analyses. Patients with hematological or oncological malignancies under chemotherapy, solid organ transplant recipients, and patients after allogeneic human stem cell transplantation were considered immunosuppressed. The cases were divided into two groups, “immunocompetent” and “immunosuppressed”, and analyzed separately.

A seasonal pattern was fitted to the data of immunocompetent patients through a logistic regression model of positive and negative test results. The seasonal curve was modelled as a cosine-function or combination of sine and cosine function across the weeks of a year. The function was shifted towards the week with maximum incidences; the amplitude of the curve was fit through the beta-estimate of the logistic model. The fit was optimized with regard to the Akaike information criterion (AIC) and predictive capacity (c-statistic, equivalent to area under the ROC). Calibration was assessed via a calibration plot (see Supplementary Materials). The *p*-value from the Hosmer–Lemeshow test (designed to reject the null hypothesis of perfect calibration) was additionally considered, with low *p*-values indicating bad model fit. The optimal scaled seasonality curve with a beta-estimate near one in immunocompetent patients was then fitted to the data of immunosuppressed patients. This provided a new beta-estimate, and the c-statistic and calibration were evaluated. In addition, the performance of this model was compared to the null model on the immunosuppressed patients through a likelihood ratio test, where low *p*-values were considered to describe good model performance. All statistical analyses were performed using SAS v.9.4 (SAS Institute, Cary, NC, USA).

Patient consent was waived due to the retrospective nature of this study. The study was conducted according to the guidelines of the Declaration of Helsinki, and approved by the Ethics Committee of the University of Duisburg-Essen (20-9265-BO, 14 April 2020).

## 3. Results

The distribution of positive cases for HMPV, PIV, RSV, and influenza throughout the year was used to evaluate potential seasonal patterns. These patterns were observed for all aforementioned pathogens, both for the immunocompetent and immunosuppressed group, to a greater or lesser degree.

### 3.1. HMPV

There were 131 HMPV-positive test results from a total of 13,580 valid measurements, originating from 6492 patients. Two positive results were excluded, as they were repeated findings of one common infection (within 28 days of a previous finding), leaving 129 and 13,578 samples, respectively, amounting to a 1% positivity rate in total.

Approximately two-thirds (n = 8862, 65.3%) of the samples originated from immunocompetent patients, of which 82 (0.9%) were positive. One-third (n = 4716, 34.7%) of the samples were from immunocompromised patients, of which 47 (1%) were positive.

**Figure 1:**
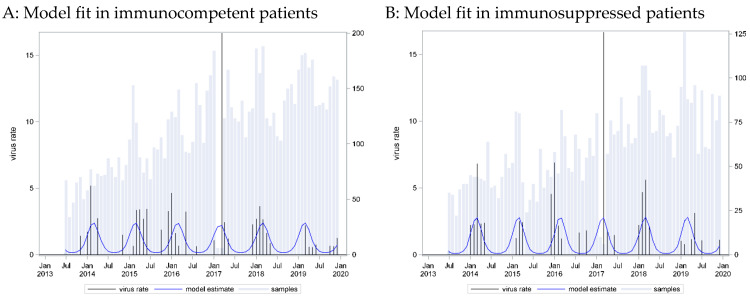
HMPV incidence rates: seasonal curve estimated for immunocompetent patients (**A**) and applied to immunosuppressed patients (**B**).

A seasonal pattern based on a cosine function with a peak in week 9 provided the best fit to these data (C = 0.73). However, application of this curve to data from immunosuppressed patients showed an equally strong association (p_LR < 0.0001) and even better predictive capacity (C = 0.76) (Figure 1).

The HMPV positivity rate was very similar in the two groups. A narrow seasonal pattern emerged as most infections were detected in the winter and early spring months, from January to March, which was similar for cases from both immunocompetent and immunocompromised patients.

### 3.2. RSV

There were 324 RSV-positive results from a total of 13,591 valid measurements. Two positive results were excluded as repeated findings of one infection (within 28 days of the previous finding), leaving 322 and 13,589 samples, respectively, amounting to an overall 2.4% positivity rate.

Approximately two-thirds (n = 8876, 65.3%) of the samples originated from immunocompetent patients, of which 217 (2.4%) were positive. One-third (n = 4713, 34.7%) of the samples were from immunocompromised patients, of which 105 (2.2%) were positive.

**Figure 2:**
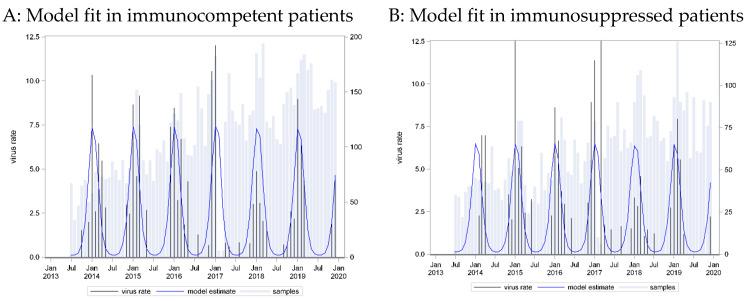
RSV incidence rates: seasonal curve estimated in immunocompetent patients (**A**) and applied to immunosuppressed patients (**B**).

A seasonal pattern based on a cosine function with a peak in week 3 provided the best fit to these data (C = 0.798). However, application of this curve to data from immunosuppressed patients showed an equally strong association (p_LR < 0.0001) and equally strong predictive capacity (C = 0.785) (Figure 2).

A wider seasonal pattern emerged. Most infections were detected in the winter and early spring months, from December to March, but occasional cases of RSV could be found throughout the year. The seasonal pattern was similar for cases from both immunocompetent and immunocompromised patients.

### 3.3. Parainfluenza (PIV)

There were 336 positive results from a total of 13,565 measurements. Four positive results were excluded as repeated findings of one infection (within 28 days of the previous finding), leaving 332 and 13,561 samples, respectively, amounting to an overall 2.5% positivity rate. In total, we detected 54 infections with PIV 1, 49 with PIV 2, 177 with PIV 3, and 59 with PIV 4, while seven patients were positive for two PIV types at the same time. As PIV types have been reported to have different seasonal distributions, we focused on subtypes 3 and 4, for which we had the most data.

For the most frequent PIV type 3, there were 8860 (65.3%) measurements available from immunocompetent patients, of which 109 (1.2%) were positive. One-third (n = 4701, 34.7%) of the cases were from immunocompromised patients, of which 68 (1.4%) were positive.

**Figure 3:**
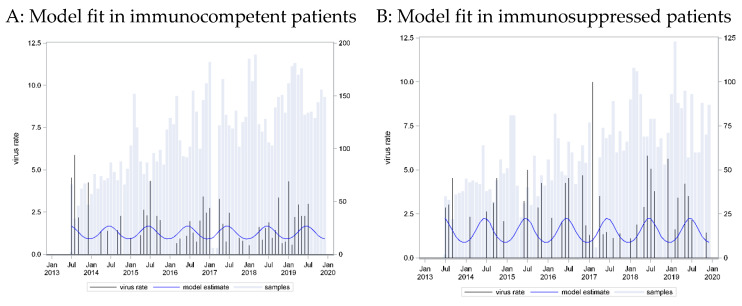
Parainfluenza virus (PIV) 3 incidence rates: seasonal curve estimated in immunocompetent patients (**A**) and applied to immunosuppressed patients (**B**).

A seasonal pattern based on a cosine function with a peak in week 25 provided the best fit to these data (C = 0.57, p_LR = 0.0188). However, application of this curve to data from immunosuppressed patients showed an equally strong association (p_LR = 0.0051) and equally strong predictive capacity (C = 0.61). Here, model calibration failed on data from immunosuppressed patients (p_Hosmer-Lemeshow = 0.0498) (Figure 3).

For PIV type 4, there were 8693 (64.1%) measurements available from immunocompetent patients, of which 40 (0.5%) were positive. One-third (n = 4595, 33.9%) of the samples were from immunocompromised patients, of which 19 (0.4%) were positive.

**Figure 4:**
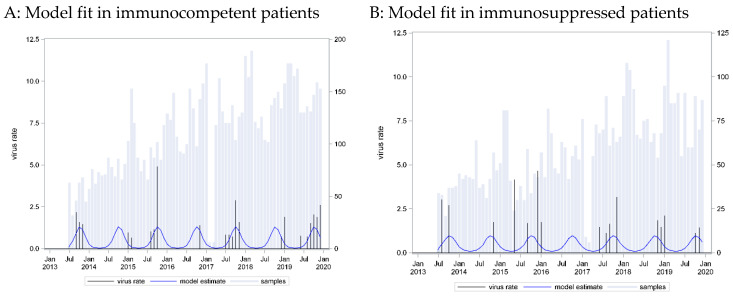
Parainfluenza virus (PIV) 4 incidence rates: seasonal curve estimated in immunocompetent patients (**A**) and applied to immunosuppressed patients (**B**).

A seasonal pattern based on a cosine function with a peak in week 42 provided the best fit to these data (C = 0.77, p_LR < 0.001). Application of this curve to data from immunosuppressed patients showed an equally strong association (beta = 0.67, p_LR = 0.0037) and equally strong predictive capacity (C = 0.70, p_Hosmer-Lemeshow = 0.3999) (Figure 4).

The positivity rate was similar in the two groups, both for PIV 3 and PIV 4. The peak week for PIV 3 was week 25 (June), while for PIV 4, it was week 42 (October), revealing a different seasonal distribution for the two subtypes. A seasonal pattern was not pronounced for PIV 3. The peak season was during the summer months, but cases could be found often throughout the year. For PIV 4, the seasonal pattern was narrower, with cases being detected mostly in Autumn. We could not detect a difference in the seasonal pattern between cases from immunocompetent and immunocompromised patients for both PIV 3 and 4.

### 3.4. Influenza Type A and Type B

For influenza type A, there were 8848 samples from immunocompetent patients, of which 219 (2.5%) were positive, while 4582 samples were generated from immunosuppressed patients, of which 98 (2.1%) were positive.

**Figure 5:**
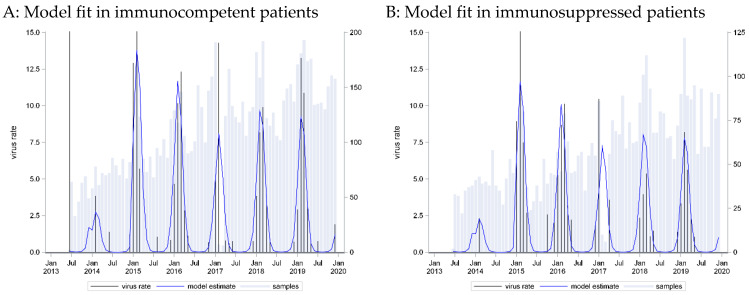
Influenza virus type A incidence rates: seasonal curve estimated in immunocompetent patients (**A**) and applied to immunosuppressed patients (**B**).

A seasonal pattern based on a cosine function with a peak in week 7 provided the best fit to these data (C = 0.87, p_LR < 0.001), and measurement year was additionally included (categories for each year). Application of this curve to data from immunosuppressed patients showed an equally strong association (beta = 0.997, p_LR < 0.0001) and equally strong predictive capacity (C = 0.87, p_Hosmer-Lemeshow = 0.8572) (Figure 5).

For influenza type B, there were 8890 samples from immunocompetent patients, of which 82 (0.9%) were positive, and 4729 from immunosuppressed patients, of which 48 (1%) were positive.

**Figure 6:**
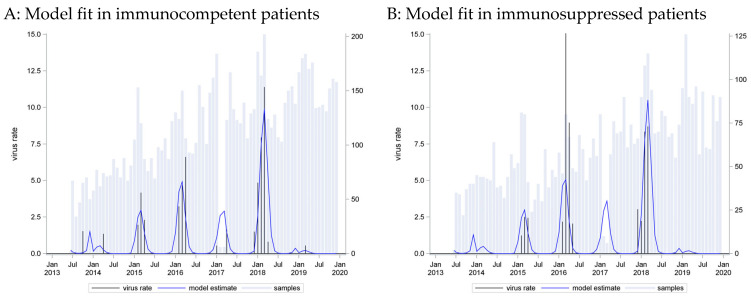
Influenza virus type B incidence rates: seasonal curve estimated in immunocompetent patients (**A**) and applied to immunosuppressed patients (**B**).

A seasonal pattern based on a cosine function with a peak in week 9 provided the best fit to these data (C = 0.91, p_LR < 0.001), and the measurement year was additionally included (categories for each year). Application of this curve to data from immune-suppressed patients showed an equally strong association (beta = 1.05, p_LR < 0.0001) and equally strong predictive capacity (C = 0.92, p_Hosmer-Lemeshow = 0.3438) (Figure 6).

The positivity rate was very similar in the two groups for both influenza types, with type A being detected more frequently than type B. A narrow seasonal pattern emerged for both types as most infections were detected in the winter, in January and February, which was similar for cases from both immunocompetent and immunocompromised patients. Additionally, we could see that the influenza type distribution differed across the different influenza seasons.

### 3.5. Immunocompromised vs. Immunocompetent: Effects of Seasonal Patterns

**Figure 7:**
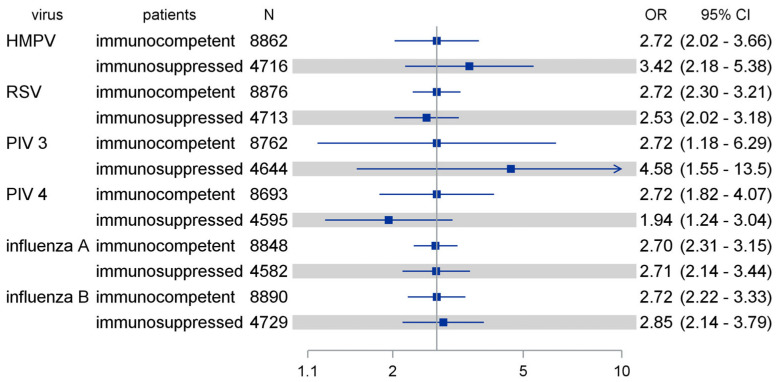
Comparing effects of seasonal patterns as optimized among immunocompetent patients in immunosuppressed patients (shaded). The seasonal calendarial pattern was optimized for logistic regression analysis to beta-coefficient = 1, corresponding to OR = exp(1) = 2.72 (reference line). HMPV: human metapneumovirus; RSV: respiratory syncytial virus; PIV: parainfluenza virus; OR: odds ratio; CI: confidence interval.

In total, when comparing the effects of seasonal patterns initially optimized for the immunocompetent cohort, we found comparable effects among immunocompromised patients, as shown in Figure 7.

## 4. Discussion

Respiratory tract infections (RTIs) are a major cause of morbidity and mortality, especially for immunosuppressed patients. Many viral RTIs follow a seasonal pattern, which varies in different geographic regions. We had previously found that the seasonal effect of a non-SARS, non-MERS coronavirus was less pronounced in immunosuppressed patients compared to immunocompetent patients, with “off-season” detection being more frequent in immunosuppressed patients [21], while a 1997 study from a USA population of immunocompromised and immunocompetent patients indicated that the pattern of occurrences of viral RTIs by time of year in immunocompromised patients are similar to those described for these viruses among immunocompetent persons, except for the frequency of RSV infection [22]. The aim of our study was to evaluate the seasonality of HMPV, PIV, influenza virus, and RSV infection in immunocompetent and immunosuppressed patients.

HMPV infections followed a narrow seasonal pattern. Most infections were detected in the winter and early spring months, from January to March. This pattern was equally pronounced in immunocompetent and immunocompromised patients. Our results concerning HMPV seasonality were largely similar to those reported in previous studies, concerning temperate climate countries. Data from the USA-based National Center for Immunization and Respiratory Diseases (NCIRD) and data from Northern China indicate that HMPV is most active during late winter and spring in temperate climates [4,20]. A previous German study showed that HMPV peaks in the winter months [15], while a study from Scotland set the high season for HMPV in March [19].

In our cohort, the most frequently detected PIV subtype was PIV 3, followed by PIV 4. The peak week for PIV 3 was week 25 (June), while for PIV 4, it was week 42 (October), revealing a different seasonal distribution for the two subtypes. A seasonal pattern was not pronounced for PIV 3. The peak season was during the summer months, but cases could be found often throughout the year. For PIV 4, the seasonal pattern was narrower, with cases being detected mostly in autumn. Results from the USA put the high season for PIV 3 in the spring and early summer months [5,23], while in a Scottish study, it was from April to May [19]. PIV 3 was most prevalent in autumn in a Korean study [24]. All PIV infections, including PIV 3, peaked in April and declined until September in Singapore, a tropical climate country [25], while a Kenyan study found no seasonal pattern of PIV infections [26]. In a South African study, PIV 3 was most prevalent in late winter and spring (July to November) [27], underlining that seasonality is dependent on geographical location and climate. The seasonal pattern of PIV 4 infection is less well described. USA data indicate that most PIV 4 infections occur in fall and winter [5], while data from South Africa indicate the high season to be late summer to early winter (February to July) [27]. We could not detect a difference in the seasonal pattern between cases from immunocompetent and immunocompromised patients for both PIV 3 and 4.

Most RSV infections in our cohort were detected in the winter and early spring months, from December to March, but cases with RSV could also be found throughout the year. The seasonal pattern was similar for cases from both immunocompetent and immunocompromised patients. This is consistent with data presented in a global overview for RSV seasonality for our region, in which the RSV high season was from December to March as well. The seasonal pattern for RSV differs in different parts of the globe, with the high season being from April to September in the Southern hemisphere [28].

For influenza, a narrow seasonal pattern emerged for both types A and B as most infections were detected in the winter (January and February), which was similar for cases from both immunocompetent and immunocompromised patients. This is largely consistent with the known seasonal patterns for influenza, which in temperate climates occur mainly during winter, while in tropical regions, influenza may occur throughout the year, causing outbreaks more irregularly [7,29].

Our study has certain limitations. We used a convenience sample, namely, patients seeking treatment at our hospital. Thus, asymptomatically infected people and the majority of mildly symptomatic individuals were not considered. Our cohort contained a very large number of immunosuppressed individuals, due to the nature of the conditions often treated in our hospital. The composition of our cohort differs from that of the general population. However, this enabled us to study the seasonality of common respiratory viral infections in immunosuppressed patients. Furthermore, the size of our sample did not allow for additional sub-analyses regarding age groups or specific medical conditions, for example, or allow for a more nuanced approach to the immunocompetence vs. immunosuppression dichotomy, since the degree of immunosuppression is not equal in all cases categorized as such. Data on the temporal pattern of common respiratory viral infection infections in immunosuppressed patients in the literature are extremely limited. Our study allows the first direct comparison of the seasonal pattern and weather effects on the HMPV, PIV, RSV, and influenza detection rates in immunosuppressed versus immunocompetent patients.

In conclusion, the seasonal pattern of HMPV, PIV, RSV, and influenza detection rate for our cohort was similar to what is expected according to its climate zone and geographic location. We could not observe any differences in the seasonal patterns of HMPV, RSV, PIV, and influenza viral infections in immunosuppressed and immunocompetent hosts.

## Data Availability

Aggregate data available upon reasonable request.

## Supplementary Materials

The following supporting information can be downloaded at https://www.mdpi.com/article/10.3390/pathogens13080704/s1, Figures S1–S6. Figure S1: HMPV-virus incidence: calibration plot of seasonal variation fit on immune competent patients (A) and applied to immune-suppressed patients (B). Figure S2: RSV incidence: calibration plot of seasonal variation fit on immune competent patients (A) and applied to immune-suppressed patients (B). Figure S3: PIV type 3: calibration plot of seasonal variation fit on immune competent patients (A) and applied to immune-suppressed patients (B). Here model calibration failed on data from immunosuppressed patients (p_Hosmer-Lemeshaw = 0.0498). Figure S4: PIV type 4: calibration plot of seasonal variation fit on immune competent patients (A) and applied to immune-suppressed patients (B). Figure S5: Influenza type A: calibration plot of seasonal variation fit on immune competent patients (A) and applied to immune-suppressed patients (B). Figure S6: Influenza type B: calibration plot of seasonal variation fit on immune competent patients (A) and applied to immune-suppressed patients (B).
