# Supplementary material for: Seasonal Patterns of Common Respiratory Viral Infections in Immunocompetent and Immunosuppressed Patients
Source: Pathogens. 2024 Aug 20;13(8):704. doi: 10.3390/pathogens13080704 (PMC11357019; doi:10.3390/pathogens13080704)

Seasonal patterns of common respiratory viral infections in immunocompetent and immunosuppressed patients.

Calibration is assessed via calibration plot. The p-value from the Hosmer-Lemeshaw test (designed to reject the Null-hypothesis of perfect calibration) is additionally provided, low p-values indicate bad model fit.

Figure S1: HMPV-virus incidence: calibration plot of seasonal variation fit on immune competent patients (A) and applied to immune-suppressed patients (B)

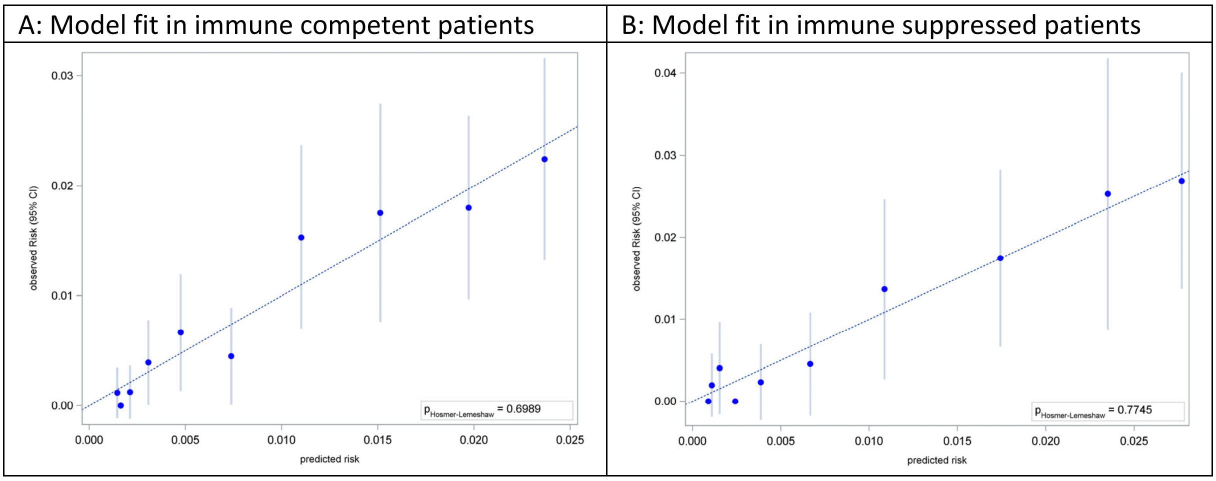

Figure S2: RSV incidence: calibration plot of seasonal variation fit on immune competent patients (A) and applied to immune-suppressed patients (B)

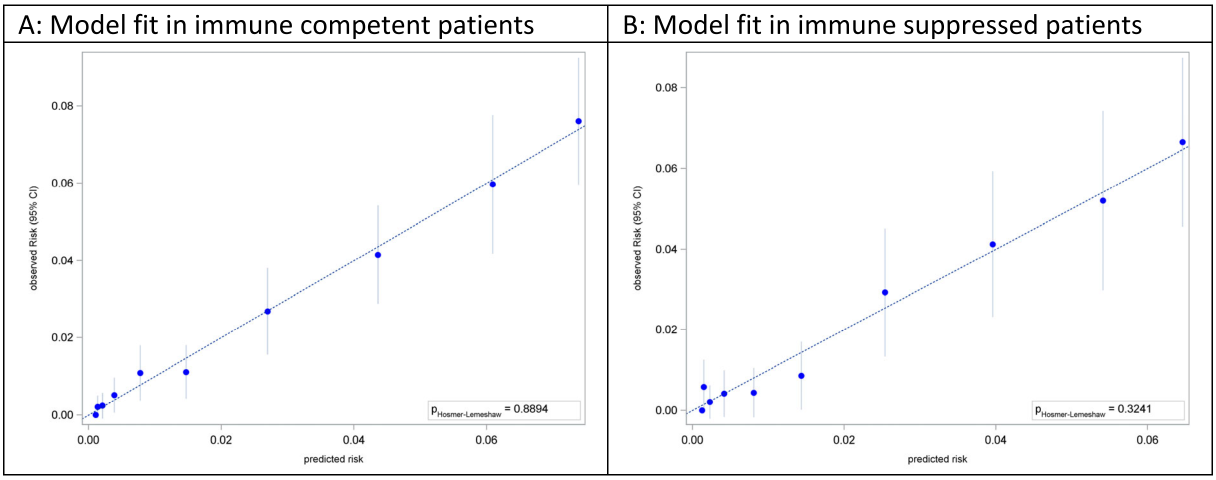

Figure S3: PIV type 3: calibration plot of seasonal variation fit on immune competent patients (A) and applied to immune-suppressed patients (B). Here model calibration failed on data from immunosuppressed patients (p_Hosmer-Lemeshaw=0.0498)

A: Model fit in immune competent patients	B: Model fit in immune suppressed patients

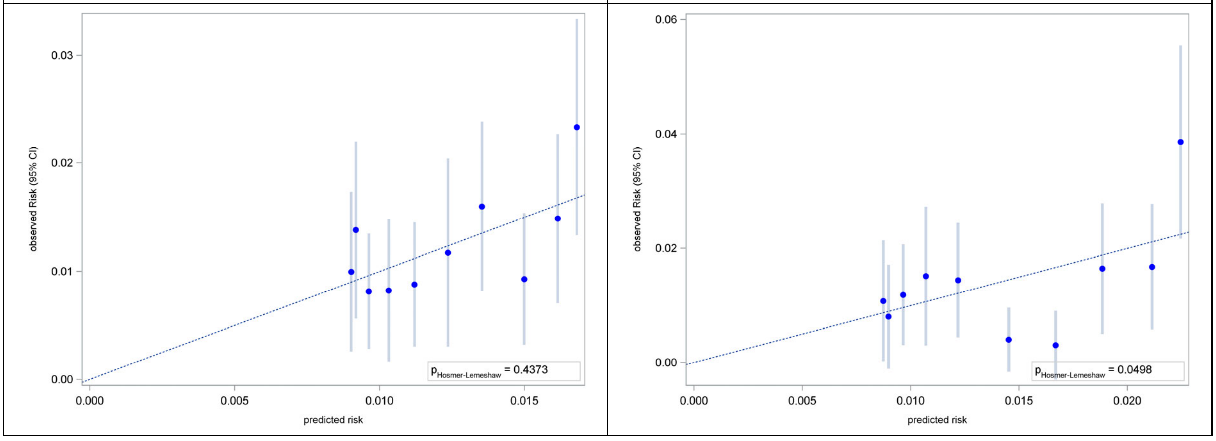

Figure S4: PIV type 4: calibration plot of seasonal variation fit on immune competent patients (A) and applied to immune-suppressed patients (B).

A: Model fit in immune competent patients	B: Model fit in immune suppressed patients

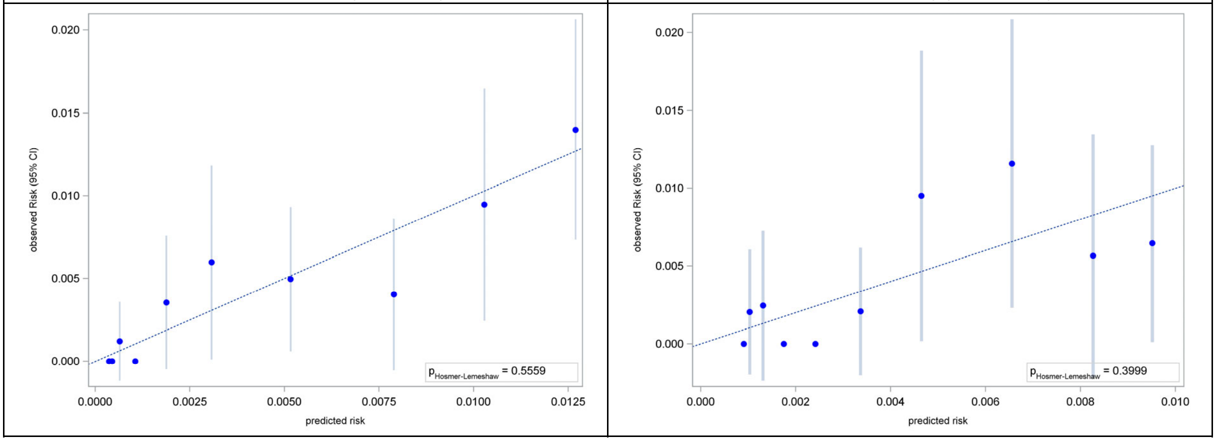

Figure S5: Influenza type A: calibration plot of seasonal variation fit on immune competent patients (A) and applied to immune-suppressed patients (B).

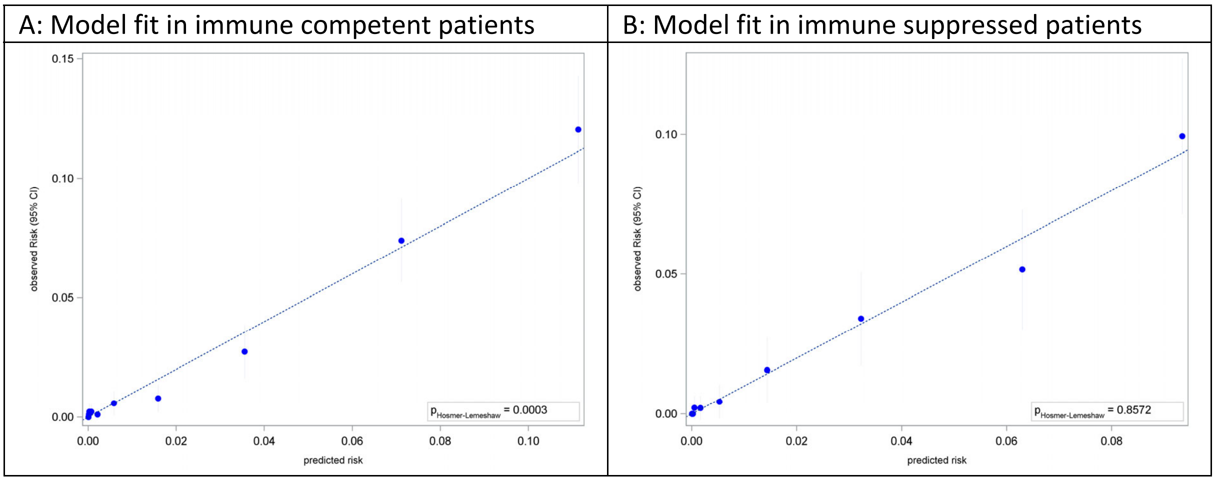

Figure S6: Influenza type B: calibration plot of seasonal variation fit on immune competent patients (A) and applied to immune-suppressed patients (B).

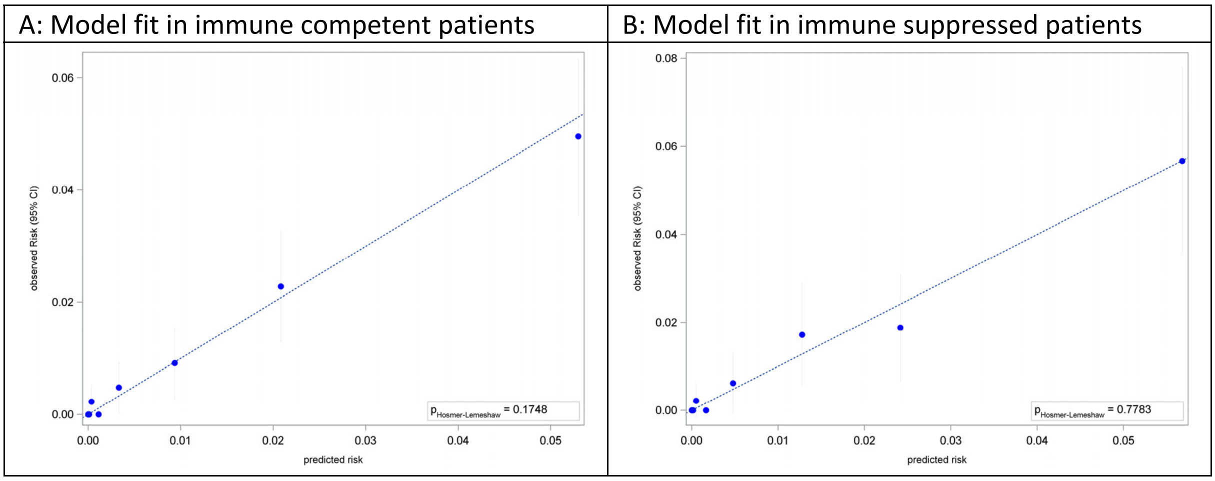

Supplement: Supplementary file 1 [file pathogens-13-00704-s001.zip › pathogens-3123288-supplementary.pdf]
